# Self-Healing Networks: Redundancy and Structure

**DOI:** 10.1371/journal.pone.0087986

**Published:** 2014-02-12

**Authors:** Walter Quattrociocchi, Guido Caldarelli, Antonio Scala

**Affiliations:** 1 Laboratory for the modeling of biological and socio-technical systems, Northeastern University, Boston, Massachusetts, United States of America; 2 LIMS the London Institute of Mathematical Sciences, Mayfair, London, United Kingdom; 3 IMT Alti Studi Lucca, Lucca, Italy; 4 ISC-CNR Uos “Sapienza”, Roma, Italy; University of Zaragoza, Spain

## Abstract

We introduce the concept of self-healing in the field of complex networks modelling; in particular, self-healing capabilities are implemented through distributed communication protocols that exploit redundant links to recover the connectivity of the system. We then analyze the effect of the level of redundancy on the resilience to multiple failures; in particular, we measure the fraction of nodes still served for increasing levels of network damages. Finally, we study the effects of redundancy under different connectivity patterns—from planar grids, to small-world, up to scale-free networks—on healing performances. Small-world topologies show that introducing some long-range connections in planar grids greatly enhances the resilience to multiple failures with performances comparable to the case of the most resilient (and least realistic) scale-free structures. Obvious applications of self-healing are in the important field of infrastructural networks like gas, power, water, oil distribution systems.

## Introduction

In the field of complex networks [1], [2], most studies have been focused on how to improve the *robustness* (i.e. the capability of surviving intentional and/or random failures) of existing networks[3]. Much less has been done regarding the *resilience* (i.e. the capability of recovering failures). In fact, implementing smart (as well as economic) strategies aimed at maintaining high level of performances is a crucial issue yet to be solved and represents one of the most pressing and interesting scientific challenge. A most important field of application for the results of such investigations are infrastructural networks. Infrastructural networks are the backbone of our society that critically depends on the continuity of functioning of systems like power, gas or water distribution.

As a standard, infrastructural networks have been designed to be resilient at least to the loss of a single component; on the other hand, their constantly growing size of has increased the possibility of multiple failures which often have not been considered in their original design. In general, implementing the possibility of recovering from *any sequence* of 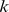 failures requires an exponentially growing effort in means and investments; it is therefore viable to consider implementing systems that are able to recover from 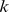 failures *on average*: in this paper we will follow such a statistical approach.

In the field of communication [4]–[6] and wireless networks [7]–[10] self-healing algorithms have recently been the subject of massive investigation. In general, such strategies aimed at maintaining network connectivity assume the possibility of creating anew communications channels among the nodes of the networks, often with no constraints on the number of new connections available [11]. This is no the case in infrastructural networks, where the possibility to create new links among nodes is normally not available (at least in the short run), since links are physical (fixed in advance) and creating new ones requires both time and investments.

In general, self-healing in infrastructural networks should be though as a constrained mechanism in which only a limited amount of resources is available. An example of such an approach can be found in material science where new polymeric compounds are capable of self healing due to the presence of small amounts of healing agents that gets released and activated upon cracking [12], [13]. An alternative strategy to ensure the continuity of a system is to ensure redundancy in the interconnectivity of its components; for example, when a hole is punched in a leaf, the remaining vessels are capable to sustain the extra flow necessary to keep the tissues alive [14].

Infrastructural networks are very well engineered systems characterised by fluxes of commodities (from electric power to drinking water). In this paper we consider a simplified description of such systems in terms of complex networks with a simple dynamical process describing the flow of a commodity from one or more sources (production) to several sinks (consumption). We the introduce a novel healing strategy based on the activation of fixed redundant resources (backup links) via a generic routing algorithm and study the resilience of the networks to multiple failures. The presence of such backup links is customary in technological networks; hence, our self-healing procedure is within the reach of current technology. As an example, urban low-voltage distribution power grids have an almost planar topology and are essentially radial (tree-like) networks with few inactive backup-links that can be activated (often manually) to restore power in case of failures.

## Results

### Model

In our scenario the system is assumed to describe a network that distributes some utility following flow conservation analogous to Kirchoff's current law; examples of systems following such constrains are not only power grids, but also the flows of fluids in distribution networks like water, gas, oil (at least at stationarity). As a further simplification, we will consider a single node to be the source of the commodity distributed on the network and we will not consider any constrain on the amount of flow that can be transported by any link; hence, connectivity among a sink and a source in enough to have the sink served. In our scenario, all the nodes (except the source) are considered to be potential sinks. Hence, to serve as many nodes as possible, connectivity must be maximized.

A further assumption is that at each instant of time, the topology of the network distributing the commodity is a tree (the *active tree*); in fact, such a structure meets the infrastructures' managers needs – i.e., to measure (for billing purposes) in an easy and precise way how much of a given quantity is served to any single node of the network. In networks – like drinking water – where such assumption is not strictly true, very few loops (i.e. low meshedness) are present [15].

To model active trees, we start from an underlying network topology and build up a spanning tree. The set of all possible redundant links is exactly the set of links in the network not belonging to the spanning tree. In order to allow for recovery, we also consider the presence of *dormant* backup links – i.e., a set of links that can be switched on – as in the case urban of low-voltage distribution power grids.

While commodities can be transported only via active links, in order to implement our self-healing strategy we assume that nodes are able to communicate by means of a suitable distributed interaction protocol only with the set of neighbouring nodes, i.e. the ones connected either via active or via dormant links. According to our procedure, when either a node or a link failure occurs, all the nodes that cannot be served – i.e. there is no path to the source – disconnect from the active tree. Afterwards, unserved nodes try to reconnect to the active tree by waking up (activating) through the protocol some of their dormant backup links. Such a process reconstructs a new active-tree that can restore totally or partially the connectivity, i.e. heals the system. A more formal description of the self-healing procedure and of the simulation protocols are provided in the Methods section.

A natural metric to quantify the success of such a procedure is the fraction of served nodes (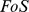). In order to identify the system's properties that are able to maximize the 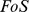 we study the effects of varying the fraction of backup links (redundancy) according to different underlying connectivity patterns with respect to multiple random failures.

In order to stress the peculiarities of different network structures, we generate class of graphs with different connectivity patterns (see Methods). We start our investigation by focusing on the underlying topology which often resembles the actual situation of infrastructural networks – i.e. nodes disposed over a planar square grid (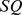). Then, we stress the role of the underlying networks' connectivity patterns by using the scale-free (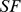) topology generated according to Barabasi-Albert [16] and the small-world (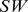) topology generated according to Watts and Strogatz[17]. All the initial network structures are generated by using the IGRAPH library [18].

To generate the random spanning trees associated to each kind of network structures, we use the flat sampling algorithm of Wilson [19]. We take such spanning trees as the initial configuration of our model distribution networks. The links not belonging to the spanning trees form the set of the possible backup links of our system; among such links, we choose a random fraction 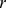 of *dormant* links that can be used to heal the system. We then simulate the occurrence of uncorrelated multiple failures by deleting at random 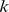 links of the initial active tree. Notice that link failures are the most general ones, as a node failure is equivalent to the simultaneous failure of all its links.

The source node – i.e, the root of the oriented active tree – is chosen at random within all the nodes of the underlying network. The only exception is the case of the 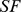 networks where we use, according to the preferential attachment principle, the natural choice of having the node with the highest number of neighbours (the central hub) as the source.

**Figure 1:**
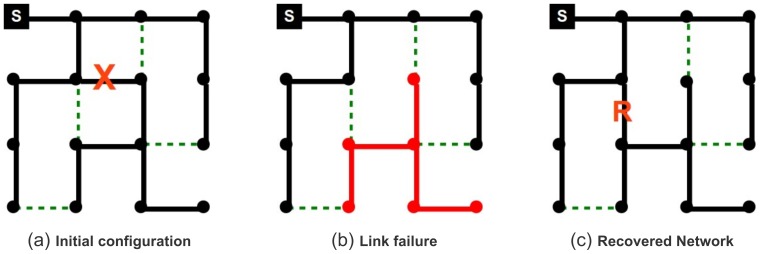
Example of healing after single link failure. Notice that failure of a single node can be modelled as the failure of all its links; hence, multiple links failure are the more general event to be considered. (Left Panel) In the initial state, the source node (filled square, upper left corner) is able to serve all 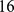 nodes through the links of the active tree. The 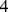 dashed lines (green online) represent dormant backup links that can be activated upon failure. The redundancy of the system is 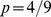 as only 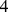 of the 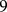 possible backup links are present. The link marked with an **X** is the one that is going to fail. (Central panel) A single link failure disconnects all the nodes of a sub-tree; in the example, a sub-tree of 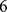 nodes (red online) is left isolated from the source – i.e., the system has a damage 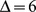). (Right Panel) By activating a single dormant backup link, the self-healing protocol has been able to recover connectivity for the whole system, in this case bringing back the number of served nodes at its maximum value 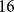. The link that has recovered the connectivity is marked with an **R**.

Our self-healing algorithm is a routing protocol (see Methods) whose goal is to reconstruct the maximum spanning tree connected to the source after that a failure has occurred; in doing so, we use both the survived links of 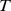 and the dormant links 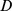; fig. 1 illustrates such procedure. After the recovery, we calculate 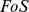 the fraction of nodes connected to the source after the recovery.

### Effects of networks' topology

In order to test the performances of our healing algorithm to failures in terms of the service provided after the active tree restoration, we simulate the model for increasing number of failures. Recalling that each failure causes a cascade – i.e, each node of sub-tree served by the broken link is unserved – we investigate the role of redundancy 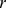 on different topologies.

**Figure 2:**
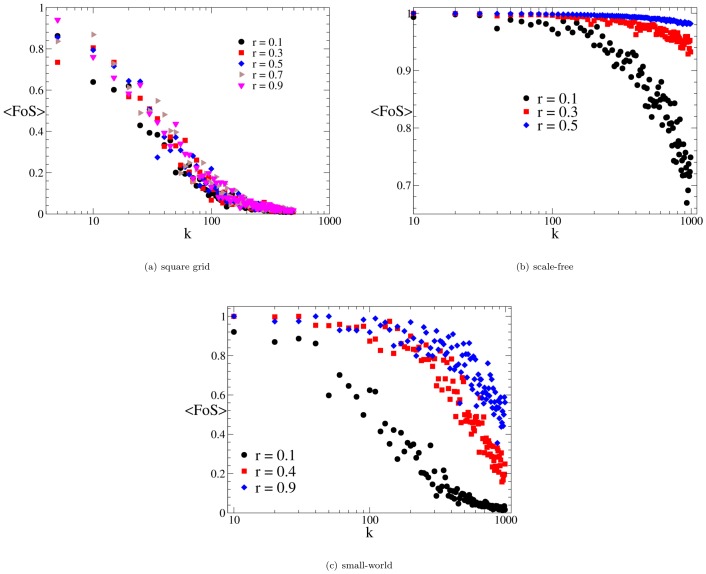
Self-healing results for networks of size 10^4^. Panel (a): distribution networks based on square grids. The average fraction 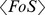 of nodes that the self-healing protocol is able to restore decreases with the number of faults 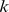 with no relevant dependency on the redundancy; results are shown for a 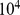 nodes network. Panel (b): distribution networks based on scale-free networks generated according to Barabasi-Albert[16]. The average fraction of nodes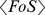 of served nodes is plotted against the number of failures 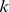. Even for a low 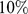 redundancy (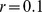), the system can almost totally heal after sustaining 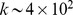 failures; as a comparison, for the same number of failures square grids loose 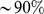 of the nodes. Panel (c): distribution grids based on small-sworld networks obtained by rewiring a fraction 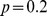 of links according to Watts-Strogatz [17]. The average fraction of nodes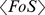 of served nodes is plotted against the number of failures 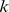. At difference with square grids and scale-free networks, the restored fraction of service 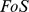 shows a marked dependency upon the redundancy parameter 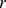. Similar results are obtained for 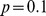 and 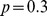.

We start our study by addressing planar square grid (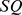) networks since they are the most similar to the real physical networked infrastructures. In the first scenario, we generate spanning trees on a square grids; fig. 2(a) shows the variation of the restored 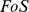 respect to the number of failures 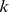 for different redundancies 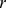
s. For square grids, we do not observe any relevance of the redundancy on the 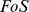; this means that a very small fraction backup links (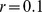, i.e. 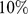) already suffice to attain the maximum resilience.

The situation is completely different when the underlying topology is a scale-free network generated through the Barabasi-Albert model [16]. A widely diffused property of real networks is that the connectivity pattern follows a scale-free power-law distribution [1], [20], [21]. This feature has been found to be a consequence of the so called *preferential attachment* – i.e networks expand continuously through the addition of new vertices which attach preferentially to already well connected nodes. Although technological networks do not show power law degree distributions due to economic and spatial constraints[22], we choose to investigate 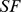 networks for their marked robustness upon random failures [23]. For 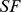 networks, it is natural to choose the node with the highest degree (the hub) as the source. The quality of service restored by our self-healing algorithm on 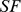 networks is shown in fig. 2(b). As expected, we find that 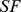 networks can easily recover connectivity to all the nodes even for low redundancies. Such error tolerance comes at a high price of being extremely vulnerable to *node* targeted attacks: isolating the hub disconnects the whole system. High error tolerance and targeted attack vulnerability are indeed generic properties of 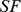 networks [24].

We then consider the case of small-world (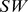) networks generated according the Watts-Strogatz rewiring procedure [17]. In the case of technological networks, small-world networks are important since they highlight the effects of introducing long-range links in a planar topology. Starting from an initial graph (planar square grids in our case), we rewire with a probability 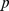 a link with a randomly selected node; in this way we can interpolate from the case of 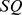 networks (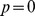) to the case of a random graph (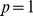). As in the case of simple percolation [25], the rewiring procedure introduces some *long range* links – i.e., between distant nodes on the square grid) that improve the robustness to random failures.

In order to understand the role of the connectivity pattern we study our model on different 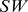 networks with different rewiring probabilities. In fig. 2(c) we show the performances of our self-healing strategy with respect to an increasing number of failures. We see that a higher rewiring probability increases the number of served nodes after the restoration through the backup network; such a peculiarity shows up even if the clustering within neighbouring nodes (normally associated to a local robustness against failures) decreases; therefore long range links increase the possibility of the network staying connected even after multiple failures.

**Figure 3:**
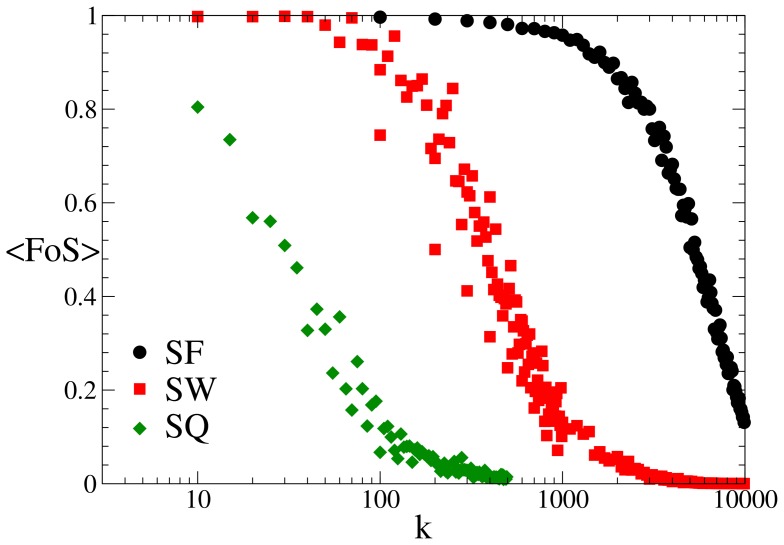
Comparison among different network structures. Here we show the performances of our self-healing algorithm with respect to the quality of service for increasing number of removed links with the redundancy 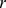 fixed to 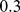; for 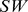 networks, the rewiring probability is 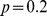. The average fraction of nodes 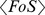 of served nodes is plotted against the number of failures 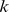.

Finally, we compare in fig. 3 the effectiveness of the self-healing protocol across different strategies. Notice that while distribution grids based on the 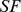 topology are the more robust, they should be disregarded when considering the case of technological networks since economic and geometric constraints make 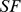 networks unfeasible on planar topologies.

## Discussion

In this paper we have introduced a minimal procedure of self-healing in networks. Such procedure exploits the presence of redundant edges to recover the connectivity of the system. Our scenario is inspired by real-world distribution networks that are – often for economic reasons – almost tree-like and at the same time are provided with alternative backup links that can be activated in case of malfunctioning. An example of such networks is the case of urban low-voltage distribution networks [26].

Our strategy could be readily and easily implemented with the current technologies. In fact, routing protocols represent a vast available source of distributed algorithms able to maintain the connectivity of a system; hence, our scheme could be implemented by the standard procedure of coupling an ICT network to a pre-existing infrastructure. Our strategy is an example in which interdependencies among two networks enhance the resilience instead of introducing catastrophic breakdowns [27].

By studying the performances of our procedure as a function of the redundancy on different underlying network topologies, we have shown that distribution networks akin to real world ones – i.e, based on planar lattices – are the less resilient to random failures. In fact, the most robust networks – as expected – are based on the 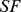 topology; however, such a topology is unrealistic for technological networks. Our results on 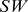 topologies hint that a very effective strategy to strengthen realistic networks is to add long range links. The feasibility of such a strategy would depend on the cost-benefit analysis about the implementation of these physical long-range links. A further direction of study would be to consider the effects of more detailed structural characteristics of the underlying network topologies [28] or even to consider biologically inspired designs, like dynamic networks inspired by the human brain [29].

While our minimal model considers only the connectivity of the system, it can be easily expanded to take account of the magnitude of the flows: in fact, routing algorithms can account for both the capacity of the links and dynamically swap re-routing of flows.

Our model easily allows also for *cold starts* – i.e., for situations in which the network has shut down due to some major events (like a black-out) [30]. This is an important issue as one of the most time (and money) consuming activity after a major event is the restoring of the functionality of the network.

In this paper, we have considered only the single source case. Next step is to consider a network served by multiple sources. In fact, the possibility of separating the system in trees would solve the *who is serving who* problem that appears as soon as more competitors share the same physical line in bringing power to their customers [31]. Moreover, the possibility for the system of dynamically separating in time-varying trees would allow for introducing a commodity market based on real-time economic competition among the owners of the sources. This further goal is not yet within the reach of current routing protocols and should be further investigated if we want to have grids that are smart not only for their ability to self-repair but also in optimizing consumptions and prices. Finally, we believe that studying and designing self-healing mechanisms in complex networks is a promising field of investigation where also the dynamics of the systems should be taken into account [32], [33].

## Materials and Methods

### The Self-Healing procedure

We consider an abstract model of a physical networked infrastructure described by the quadruple 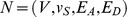. Here 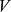 are nodes of the network, 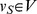 is the source node, 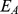 is the set of active links among the nodes and 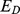 denotes the set of dormant links that can be activated in order to heal system failures by re-connecting nodes. A node is considered to be served if it is connected to a source through a path of active links; all the nodes in 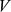 are initially connected to the source via a spanning tree. As the basic metric for any quality of service assessment, we consider the fraction of served nodes 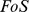 counting the number of nodes in the active graph – i.e connected to 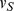.

More formally, in the initial configuration, the graph 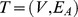 is an instance of the set 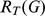 of all the random spanning tree of the underlying graph 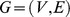. Thus, 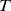 before the failures has 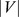 (active) nodes and 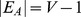 links among them. The set 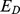 of backup edges is taken form the remaining edges of the underlying graph 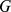, i.e. 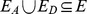 and 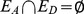. The fraction 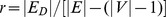 measures the redundancy of 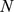.

We then consider the occurrence of multiple link failures. A 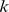-failure is a subset 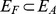 of 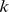 links chosen at random. The system right after a failure is described by the forest 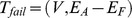 and by the set 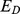 of dormant links available for the healing. A healing protocol is any algorithm that, by activating (waking up) a subset 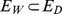 of dormant edges, finds a maximal tree 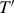 of 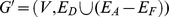 containing the source 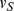. If 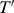 is spanning, then the system has fully recovered.

For the robustness of the algorithm, we will assume that nodes have only a *local* knowledge of the networks – i.e., only about the state (active, dormant or failed) of their incoming links. To build the maximal connected tree, nodes communicate with their neighbors via a suitable distributed protocol allowing fault nodes to join the active network by activating *dormant* edges. In other words, nodes are endowed only with the minimal requirements of routing needed to reconstruct a spanning tree [34]. In this paper, we have applied the following simple distributed algorithm to implement self-healing:

   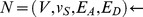 initial configuration

   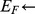 failed links

   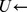 unserved nodes

   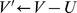


   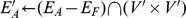


   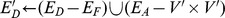


   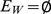


   **repeat**

      **for all** 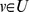 **do**

         choose a random neighbor 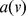 connected to 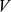 through any edge of 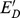


      **for all** 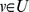 **do**

         **if** 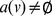 **then**

            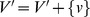


            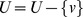


            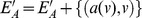


            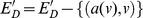


            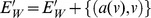


   **until** 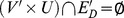


   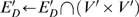


   **return** 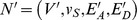


By definition, the nodes in 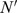 are the set of served nodes 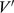. Notice that the state 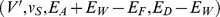 still describes a network infrastructure; therefore, we can in general describe the state of the system at time 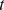 by the quadruple 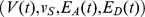 and the sequence of time failures between time 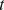 and 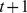 by 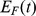. A general representation of such a process can be given in terms of time varying graph [35].

### Simulations

**Figure 4:**
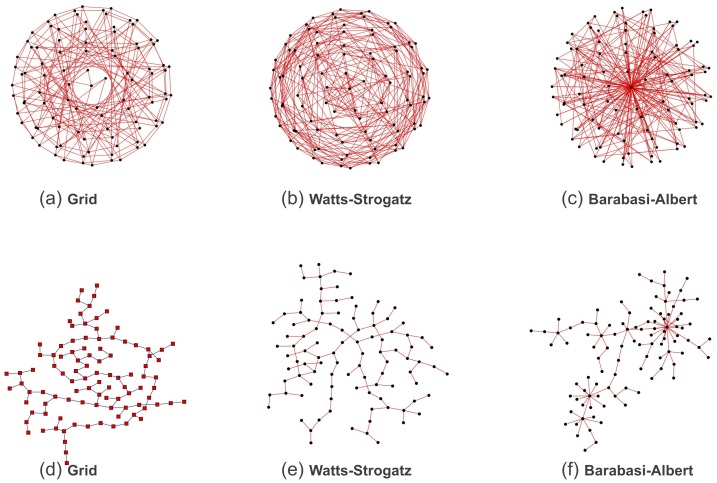
Different network topologies. Upper panels, from left to right: planar square grid (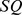), small-world network (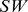) generated according to Watts-Strogatz [17] and scale-free network (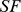) generated accordint to Barabasi-Albert [16]. Lower panels: random spanning trees associated with the related underlying topologies in the upper panels.

We analyse the response of the system to 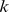-failures. To study the effects of different topologies, we perform simulations on different grids (fig.4 – upper panel).

Classically, engineered systems (especially in the power industry) are built 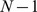 robust, i.e. they can survive to the single failure of any of their components. While checking 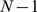 robustness corresponds to checking all the 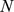 possible single failures, checking the 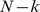 robustness requires to consider 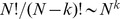 cases. Therefore, checking the 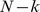 robustness is infeasible even for modest values of 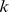 due to the combinatorial explosion of the number of possible cases. Thus, we choose to assess on probabilistic ground whether a system would be able to sustain 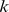 failures by a Monte Carlo investigation of the space of possible failures.

Service operators are interested in maintaining their service level agreements (contracts) with their customers; to such an aim, customers must in first pace remain connected to the services. Therefore, we calculate the average fraction of served customers 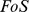 after the occurrence and the healing of 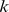 random failures. To do so, we choose at random 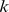 different links on the service tree and delete them; after that, we apply the self healing procedure; finally, we calculate the 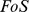 as the fraction of nodes connected to the source. We average such procedure over several network realizations until the relative error of the average 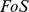 is small enough (less than 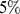). As an example, for a grid of 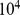 nodes, we must typically average over 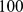 sets of random failures to attain the desired accuracy. Moreover, to average out the different characteristics of the initial configurations, we repeat the procedure over 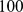 different independently generated initial configurations.

To generate a random spanning tree 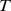 associated to a graph 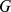 (fig.4 – lower panel), we apply the exact algorithm of Wilson [19] that samples uniformly the elements of 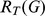. Such spanning trees are taken as the initial configurations for our model distribution networks. The links of the graph 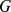 that do not belong to the initial configuration 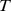 form the set 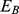 of the possible backup links of our system; of such links, only a subset 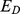 (the *dormant* links) can be used to heal the system. The fraction 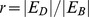 of such dormant links characterizes the redundancy of the system: for 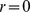 there are no links in 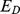 and any failure splits the tree, while for 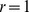 any of the links of 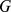 can be used to recompose the system.

Notice that in our case it would be more correct to speak about 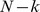 *resilience*, since we don't consider whether the system is *robust* to 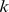 failures (i.e. whether it still functioning after 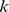 failures), but if it can *recover* from 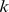 failures.

## References

[1] Caldarelli G (2007) Scale-Free Networks: Complex Webs in Nature and Technology (Oxford Finance). Oxford University Press, USA. Available: http://www.amazon.ca/exec/obidos/redirect?tag=citeulike09-20\\&path=ASIN/0199211515.

[2] Barrat A, Barthélemy M, Vespignani A (2008) Dynamical Processes on Complex Networks. Cambridge University Press. Available: http://books.google.it/books?id=TmgePn9uQD4C.

[3] Schneider CM, Moreira AA, Andrade JS, Havlin S, Herrmann HJ (2011) Mitigation of malicious attacks on networks. Proceedings of the National Academy of Sciences 108: 3838–3841. 10.1073/pnas.1009440108 PMC3053993 21368159

[4] Flocchini P, Pagli L, Prencipe G, Santoro N, Widmayer P (2008) Computing all the best swap edges distributively. J Parallel Distrib Comput 68: 976–983.

[5] Velazquez E, Santoro N (2010) Mobility-based strategies for energy restoration in wireless sensor networks. In: MSN. pp. 161–168.

[6] Flocchini P, Enriquez TM, Pagli L, Prencipe G, Santoro N (2012) Distributed minimum spanning tree maintenance for transient node failures. IEEE Trans Computers 61: 408–414.

[7] Sarma AD, Trehan A (2012) Edge-preserving self-healing: Keeping network backbones densely connected. In: INFOCOM Workshops. pp. 226–231.

[8] Kawamura R, Sato KI, Tokizawa I (2006) Self-healing atm networks based on virtual path concept. IEEE JSel A Commun 12: 120–127.

[9] Murakami K, Kim HS (1998) Optimal capacity and flow assignment for self-healing atm networks based on line and end-to-end restoration. IEEE/ACM Trans Netw 6: 207–221.

[10] Ramchurn SD, Vytelingum P, Rogers A, Jennings NR (2012) Putting the ‘smarts’ into the smart grid: a grand challenge for artificial intelligence. Commun ACM 55: 86–97.

[11] Pandurangan G, Trehan A (2011) Xheal: localized self-healing using expanders. In: PODC. pp. 301–310.

[12] White SR, Sottos NR, Geubelle PH, Moore JS, Kessler MR, et al (2001) Autonomic healing of polymer composites. Nature 409: 794–797. 11236987 10.1038/35057232

[13] Toohey KS, Sottos NR, Lewis JA, Moore JS, White SR (2007) Self-healing materials with microvascular networks. Nat Mater 6: 581–585. 17558429 10.1038/nmat1934

[14] Katifori E, Szöllősi GJ, Magnasco MO (2010) Damage and fluctuations induce loops in optimal transport networks. Phys Rev Lett 104: 048704. 20366746 10.1103/PhysRevLett.104.048704

[15] Yazdani A, Jeffrey P (2011) Complex network analysis of water distribution systems. Chaos: An Interdisciplinary Journal of Nonlinear Science 21: 016111. 10.1063/1.3540339 21456853

[16] Barabási AL, Albert R (1999) Emergence of scaling in random networks. Science 286: 509–512. 10521342 10.1126/science.286.5439.509

[17] Watts DJ, Strogatz SH (1998) Collective dynamics of /‘small-world/’ networks. Nature 393: 440–442. 9623998 10.1038/30918

[18] Csardi G, Nepusz T (2006) The igraph software package for complex network research. InterJournal Complex Systems: 1695.

[19] Wilson DB (1996) Generating random spanning trees more quickly than the cover time. In: Proceedings of the 28th annuan ACM Symposium on the Theory of Computing. ACM, pp. 296–303.

[20] Pastor-Satorras R, Vespignani A (2001) Epidemic spreading in scale-free networks. Phys Rev Lett 86: 3200–3203. 11290142 10.1103/PhysRevLett.86.3200

[21] Aoki T, Aoyagi T (2012) Scale-free structures emerging from co-evolution of a network and the distribution of a diffusive resource on it. Phys Rev Lett 109: 208702. 23215528 10.1103/PhysRevLett.109.208702

[22] Amaral LAN, Scala A, Barthélémy M, Stanley HE (2000) Classes of small-world networks. Proceedings of the National Academy of Sciences 97: 11149–11152. 10.1073/pnas.200327197 PMC17168 11005838

[23] Bollobas B, Riordan O (2004) Robustness and vulnerability of scale-free random graphs. Internet Mathematics 1: 1–35.

[24] Albert R, Jeong H, Barabasi AL (2000) Error and attack tolerance of complex networks. Nature 406: 378–382. 10935628 10.1038/35019019

[25] Newman MEJ, Watts DJ (1999) Scaling and percolation in the small-world network model. Phys Rev E 60: 7332–7342. 10.1103/physreve.60.7332 11970678

[26] ENEL (2011) private communication.

[27] Buldyrev SV, Parshani R, Paul G, Stanley HE, Havlin S (2010) Catastrophic cascade of failures in interdependent networks. Nature 464: 1025–1028. 20393559 10.1038/nature08932

[28] D'Agostino G, Scala A, Zlatić V, Caldarelli G (2012) Robustness and assortativity for diffusionlike processes in scale-free networks. EPL 97: 68006.

[29] Yuan WJ, Zhou JF, Li Q, Chen DB, Wang Z (2013) Spontaneous scale-free structure in adaptive networks with synchronously dynamical linking. Phys Rev E 88: 022818. 10.1103/PhysRevE.88.022818 24032894

[30] Sudhakar TD, Srinivas KN (2011) Restoration of power network? a bibliographic survey. European Transactions on Electrical Power 21: 635–655.

[31] Li H, Zhang W (2010) Qos routing in smart grid. In: Global Telecommunications Conference (GLOBECOM 2010), 2010 IEEE. pp. 1–6. doi:10.1109/GLOCOM.2010.5683884.

[32] Boccaletti S, Latora V, Moreno Y, Chavez M, Hwang DU (2006) Complex networks: Structure and dynamics. Physics Reports 424: 175–308.

[33] Arenas A, Diaaz-Guilera A, Kurths J, Moreno Y, Zhou C (2008) Synchronization in complex networks. Physics Reports 469: 93–153.

[34] Santoro N (2006) Design and Analysis of Distributed Algorithms (Wiley Series on Parallel and Distributed Computing). Wiley-Interscience.

[35] Casteigts A, Flocchini P, Quattrociocchi W, Santoro N (2012) Time-varying graphs and dynamic networks. 27: 387–408.

